# Validation and analysis of the geographical origin of *Angelica sinensis* (Oliv.) Diels using multi-element and stable isotopes

**DOI:** 10.7717/peerj.11928

**Published:** 2021-08-06

**Authors:** Shanjia Li, Hui Wang, Ling Jin, James F. White, Kathryn L. Kingsley, Wei Gou, Lijuan Cui, Fuxiang Wang, Zihao Wang, Guoqiang Wu

**Affiliations:** 1 School of Life Science and Engineering, Lanzhou University of Technology, Lanzhou, Gansu, China; 2 Key Laboratory of Land Surface Process and Climate Change in Cold and Arid Regions, Northwest Institute of Eco-Environment and Resources, Chinese Academy of Sciences, Lanzhou, Gansu, China; 3 College of Pharmacy, Gansu University of Chinese Medicine, Lanzhou, Gansu, China; 4 Department of Plant Biology, Rutgers University, New Brunswick, United States of America

**Keywords:** *Angelica sinensis*, Mineral elements, Stable isotopes, Discriminant analysis

## Abstract

**Background:**

Place of origin is an important factor when determining the quality and authenticity of* Angelica sinensis* for medicinal use. It is important to trace the origin and confirm the regional characteristics of medicinal products for sustainable industrial development. Effectively tracing and confirming the material’s origin may be accomplished by detecting stable isotopes and mineral elements.

**Methods:**

We studied 25 *A. sinensis* samples collected from three main producing areas (Linxia, Gannan, and Dingxi) in southeastern Gansu Province, China, to better identify its origin. We used inductively coupled plasma mass spectrometry (ICP-MS) and stable isotope ratio mass spectrometry (IRMS) to determine eight mineral elements (K, Mg, Ca, Zn, Cu, Mn, Cr, Al) and three stable isotopes (δ^13^C, δ^15^N, δ^18^O). Principal component analysis (PCA), partial least square discriminant analysis (PLS-DA) and linear discriminant analysis (LDA) were used to verify the validity of its geographical origin.

**Results:**

K, Ca/Al, δ^13^C, δ^15^N and δ^18^O are important elements to distinguish *A. sinensis* sampled from Linxia, Gannan and Dingxi. We used an unsupervised PCA model to determine the dimensionality reduction of mineral elements and stable isotopes, which could distinguish the *A. sinensis* from Linxia. However, it could not easily distinguish *A. sinensis* sampled from Gannan and Dingxi. The supervised PLS-DA and LDA models could effectively distinguish samples taken from all three regions and perform cross-validation. The cross-validation accuracy of PLS-DA using mineral elements and stable isotopes was 84%, which was higher than LDA using mineral elements and stable isotopes.

**Conclusions:**

The PLS-DA and LDA models provide a theoretical basis for tracing the origin of *A. sinensis* in three regions (Linxia, Gannan and Dingxi). This is significant for protecting consumers’ health, rights and interests.

## Introduction

*Angelica sinensis* is a native Chinese plant (Ross, 2001). Its dried root, commonly known as Danggui in China, is frequently used in Chinese traditional medicines. Its use was first recorded in the Divine Farmer’s Classic of Materia Medica (also known as Shennong Bencao Jing) more than two thousand years ago (Ai et al., 2013). It is widely distributed in China, Korea, Japan, Europe and America (Lu et al., 2020; Mei et al., 2015; Zhao et al., 2003). The Dingxi region in the Gansu Province of China is the primary *A. sinensis* production area, accounting for 70% of China’s total production and 80% of its export (Giacomelli et al., 2017). *A. sinensis* is often used to treat gynecological diseases (Circosta et al., 2006) and is also called “female ginseng” (Hook Ingrid, 2014; Tian et al., 2017). *A. sinensis* is becoming increasingly popular worldwide as a health supplement for women.

Recently, food and drug safety issues have drawn much attention due to increased global import and export trade. However, we lack effective scientific tools and techniques to ensure the enforcement of laws and regulations (Tang et al., 2015). The geographical origin of *A. sinensis* has been unexplored so far. Previous research has mainly focused on the extraction and separation of natural compounds from *A. sinensis* and screening their bioactivity (Chao & Lin, 2011; Jin et al., 2012). More than 70 compounds have been isolated from the dried roots of *A. sinensis* (Chao & Lin, 2011), of which ferulic acid and Z-lignoside are the two main identified components (Giacomelli et al., 2017; Lu et al., 2005). The molecular genetics of *A.sinensis* are not well studied (Mei et al., 2015; Zhao et al., 2003). Molecular genetics can explain the genetic distance between *A. sinensis* varieties, but it cannot be used as evidence of geographic distance (Adamo et al., 2012). Thus our ability to trace its geographic origin is limited.

Many countries have issued laws and regulations for food and drug traceability, resulting in the development of radio frequency identification (RFID), barcode readers (George et al., 2019) and two-dimensional barcode technology (Chen et al., 2020). Although these technologies can track food source and flow, it is only limited to a document label description. It is impossible to guarantee whether the label matches the product in complex product transportation and processing chains. Furthermore, the provenance of food traceability must also be verified. The geographical tracing of food and drugs typically occurs through mineral elements (Coelho et al., 2019; Sayago, González-Domínguez & Beltrán, 2018; Potortì et al., 2018), stable isotopes (Camin et al., 2017; Pianezze et al., 2019; Wadood, Guo & Wei, 2019; Zhou et al., 2019), organic compounds (Lukić et al., 2018; Fang et al., 2019; Liu et al., 2017), and infrared spectral absorption characteristic peaks (Wang et al., 2019; Hu et al., 2019; Innamorato et al., 2019). Tracing based on mineral elements and stable isotopes has been used for a variety of plant and animal products with geographical origin traceability. For example, tea (Deng et al., 2019; Zhang et al., 2018), pear (Albergamo et al., 2018), rice (Liu et al., 2018), olive oil (Damak et al., 2019), alcohol (Geană et al., 2017; Pepi et al., 2019; Wu et al., 2019), meat (Hao et al., 2019) and aquatic products (Han et al., 2020; Luo et al., 2019; Li et al., 2018) have been traced using these methods.

Mineral elements in soil are significantly related to corresponding levels in plants, in respect of the traceability of agricultural raw materials (Greenough, Mallory-Greenough & Fryer, 2005; Brzezicha-Cirocka, Grembecka & Szefer, 2016; Catarino et al., 2008). The mineral elements in *A. sinensis* reflect its contact with soil geochemistry. The regional geological heterogeneity could lead to variations in the mineral-element contents of *A. sinensis*. Mineral elements are mostly stable in the raw materials of agricultural products compared with other components, which makes this a promising identifier for determining food provenance (Zhao, Zhang & Zhang, 2017; Catarino et al., 2018). Stable isotopes of plants are affected by environmental factors and its temporal and spatial specificity. It has been reported that δ^13^C and δ^18^O are closely related to factors such as precipitation, temperature, altitude, and slope (Hultine & Marshall, 2000; Granath et al., 2018). Among them, δ^13^C values depend on the relative CO_2_ value in the external environment and the CO_2_ concentration of the intercellular environment (mainly stomatal density and stomatal conductance) (Ferrio, Voltas & Araus, 2003). δ^18^O values in plants mainly depend on atmospheric precipitation and transpiration (Ripullone et al., 2008). Precipitation and radiation show temporal and spatial specificity, which leads to differences in the δ^18^O enrichment in plants. δ^15^N values in plants are mainly affected by nitrogen pools in soil. δ^15^N typically exists in the form of nitrogen compounds. The nitrogen compounds from different soil types lead to variations of δ^15^N across different geographical regions through nitrification, denitrification and ammonia volatilization (Handley & Raven, 2010). Therefore, the spatial distribution of stable isotope ratios in the environment is necessary to determine the *A. sinensis*’ isoscapes. This study provides a theoretical basis for evaluating the geographical origin of *A. sinensis* based on the calibrated characteristic values of a stable isotope.

In this study, 25 samples of *A. sinensis* were collected from three regions (Linxia, Gannan, and Dingxi) in the southeastern province of Gansu, China. We measured three stable isotopes and eight mineral elements. Principal component analysis (PCA), partial least square discriminant analysis (PLS-DA) and linear discriminant analysis (LDA) for multivariate data discriminant analysis were conducted. We sought to (1) describe the differences between stable isotopes and mineral elements in three different regions (Linxia, Gannan and Dingxi); (2) screen the landmark mineral elements and stable isotope factors of *A. sinensis* in the different regions; and (3) compare PCA, PLS-DA and LDA’s discrimination and cross-validation ability on *A. sinensis*.

## Materials & Methods

### Study area and sample collection

Samples were taken from the Linxia (LX), Gannan (GN) and Dingxi (DX) regions of southeast Gansu Province, China, between 34°24′–35°57′N and 103°11′–104°28′E. Linxia has a semi-arid climate, with an average annual temperature of 5.2–7.0 °C and an average annual precipitation of 350–660 mm. Gannan has a plateau climate, with large regional differences in annual average temperature and extremely uneven geographical distribution of precipitation; the average annual temperature is between 1–13 °C and the average annual precipitation is 518–634 mm. Dingxi is a middle temperate semi-arid area with a continental monsoon climate, with an average annual temperature of 5.5 °C and an average annual precipitation of 635 mm.

**Figure 1:**
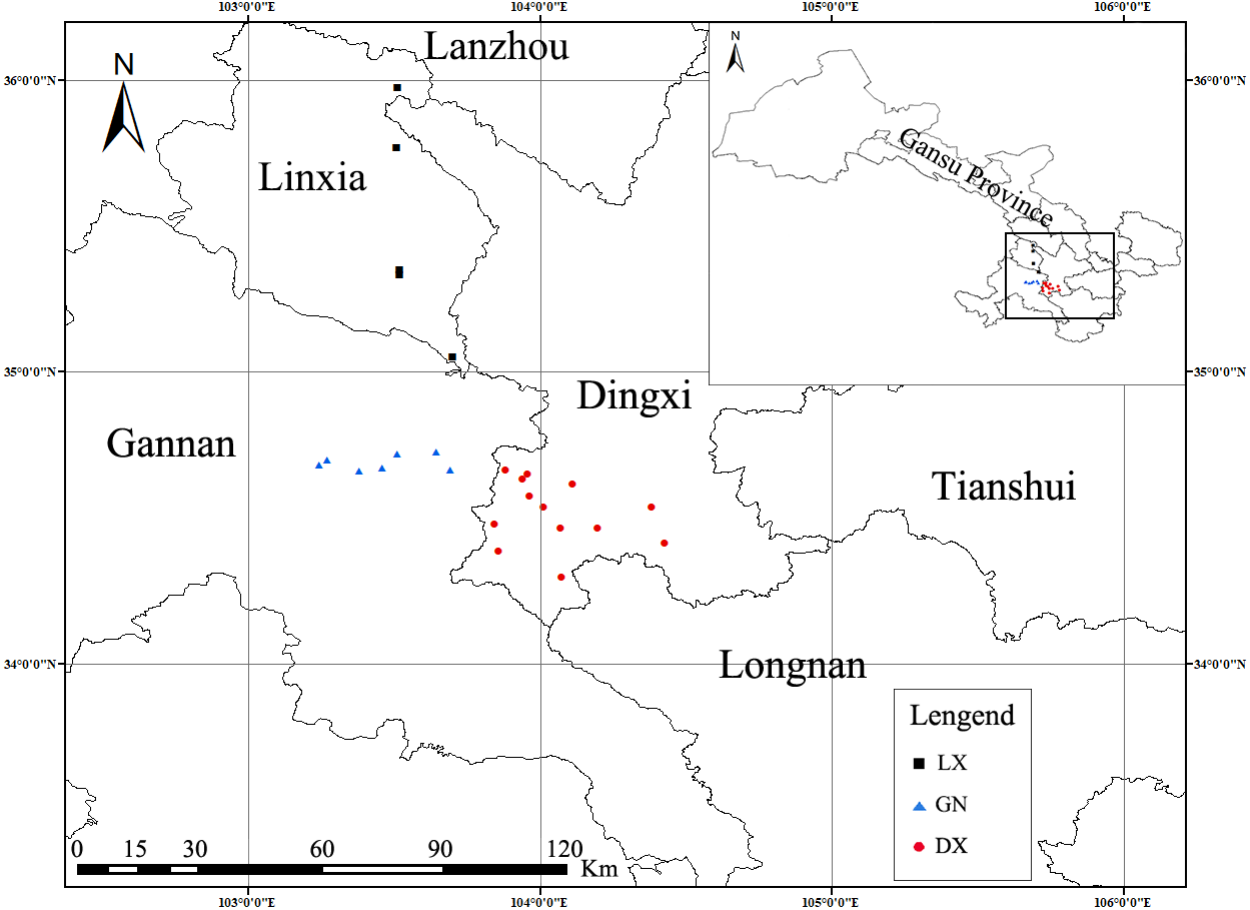
Geographical distribution of *A. sinensis* sampling areas in three regions (Linxia, Gannan and Dingxi).

We collected 25 samples from farmlands during the *A. sinensis* harvest season from April to May 2019. We contacted local farmers to obtain their permission to collect experimental samples. Global Positioning System (GPS) was used to record the longitude and latitude of each sampling plot, and ArcGIS (Version 10.7) was used to plot the sampling points. As shown in Fig. 1, samples were collected from planting sites (including five in Linxia, seven in Gannan, and 13 in Dingxi). We dug the roots of *A. sinensis*, gently brushed away the surface soil, and stored the sample in a self-sealing bag. Samples were put into a 4 °C fresh-keeping container for refrigeration. A freshly collected sample was cleaned and rinsed with deionized water three times then dried in a constant temperature oven at 70 °C to a constant weight. Dried roots were crushed by a high-speed pulverizer and were passed through a 100-mesh sieve. These samples were placed in self-sealing bags for storage.

### Stable isotope measurement

We weighed 5.0 mg of ground *A. sinensis* into tin capsules. We used an isotope ratio mass spectrometer (IRMS Delta plus XP; Thermo-Fisher, USA) and an element analyzer (Flash EA) to analyze δ^13^C and δ^15^N. The sample was introduced into the element analyzer through the autosampler and was transferred to the IRMS through the carrier gas (helium) to determine δ^13^C and δ^15^N. Reference materials, including USGS24, IAEA-600, IAEA-N-2 and IAEA-NO-3, were used for calibration. During the analysis, a laboratory standard was interspersed for every 12 samples for calibration. The long-term accuracy of the instrument was 0.2‰. We used a known laboratory wheat flour (B2159) standard with δ^13^C (δ^13^C_V−PDB_ =−13.68 ± 0.2 (‰)) and δ^15^N (δ^15^N_Air_ =1.58 ± 0.15 (‰)) values to check the accuracy of the instrumental condition. The samples were analyzed in triplicate. We calculated the average of the three samples.

In order to perform δ^18^O analysis, we weighed 1 mg of the dried *A. sinensis* sample and packed it into an isotope silver capsule. Samples were loaded into the elemental analyzer Flash EA through the autosampler, and the analyte was sent to the isotope ratio mass spectrometer (253plus; Thermo-Fisher, USA) for δ^18^O determination. The international reference standards for δ^18^O are IAEA-601and USGS55. The samples were analyzed in triplicate. We calculated the average of the three samples.

The following formula was used to calculate the relative deviation of the measured δ^13^C, δ^15^N and δ^18^O from the ratio of international standard substances:

\begin{eqnarray*}\mathrm{\delta } \left( \permil \right) = \left[ \frac{{R}_{x}-{R}_{std}}{{R}_{std}} \right] \times 1000 \end{eqnarray*}

—δ(‰): The heavier isotope values in the sample (^13^C, ^15^N, ^18^O);

R_x_: The ratio of stable isotope in the sample (^13^C/^12^C, ^15^N/^14^N, ^18^O/^16^O);

R_std_: International standard material stable isotope ratio ((^13^C/^12^C)_VPDB_, (^15^N/^14^N)_Air_, (^18^O/^16^O)_VSMOW_)

### Mineral elements analysis

All of the materials used for standard solution and sample processing and the Teflon digestion vessel were immersed in 20% HNO_3_ solution for 24 h, then rinsed with deionized water and dried before testing the standard solution and samples. We used inductively coupled plasma mass spectrometry (ICP-MS) tuning solution to calibrate ICP-MS. The Rh standard solution was used as an internal standard. We prepared the working standard solution by diluting the stock standard solution of K, Mg, Ca, Zn, Cu, Mn, Cr and Al with 2% HNO_3_. The preparation concentration of K, Mg and Ca was 0–150 mg/L, and the preparation concentration of Zn, Cu, Mn, Cr and Al was 0–500 µg/L. Each working standard solution tested was repeated three times to obtain a standard curve. The standard curve R^2^ of all the elements measured was greater than 0.9990.

We weighed 0.3 ± 0.01 g of the *A. sinensis* powder. These samples were then placed into a microwave digestion tank and 8 mL of concentrated HNO_3_ was added. The container was covered and digested. After digestion was complete, the *A. sinensis* sample was cooled to room temperature in a 25 mL volumetric flask to a constant volume, and this was repeated three times. The digestion process was: (1) 800 W constant microwave power at 100 °C for 10 min; (2) 800 W constant microwave power at 150 °C for 10 min; (3) 800 W constant microwave power at 180 °C for 30 min.

ICP-MS was used to determine eight mineral elements in *A. sinensis* samples (Albergamo et al., 2018) and their quantification limits were 0.003 mg/L (K), 0.008 µg/L (Ca), 0.0008 mg/L (Mg), 2.830 µg/L (Al), 0.062 µg/L (Zn), 0.023 µg/L (Mn), 0.443 µg/L (Cu), and 0.026 µg/L (Cr). The limit of quantification (LOQ) was the analyte concentration equivalent to 10 times the standard deviation (10 *σ*) of the blank solution for 10 consecutive measurements.

### Statistical analysis

We analyzed the significant differences between variables form the three different regions after testing the 11 variables’ distribution and the analysis variables’ basic statistics. These were statistically analyzed by grouping (*P* < 0.05), and multiple comparisons between paired groups were performed by using Tukey’s honestly significant differences (Tukey HSD) method. We standardized the data and performed dimensionality reduction and discriminant analysis.

The traceability and provenance of food depends on reliable measurement data and using the appropriate chemometric data processing methods (Bertacchini et al., 2013). Single element stoichiometry is greatly limited in terms of traceability, and it is impossible to comprehensively evaluate a large number of variables with it. Thus, a lot of important traceability information is lost. Multivariate data analysis techniques such as PCA, PLS-DA, and LDA can effectively realize the comprehensive analysis of multiple “fingerprint” information. PCA analysis is an internal exploration of data, with only a natural grouping of a multivariate data set and no predictive function. PLS-DA is a supervised discrimination method and is different from PCA. It can integrate the basic functions of multiple linear regression analysis, canonical correlation analysis and principal component analysis and can solve the multicollinearity problem in linear regression (Brereton & Lloyd, 2014). PLS-DA assigns groups to the samples before classification. After grouping, the model adds an implicit virtual binary matrix as the response sample category (specifying a group as 1, and all other values are 0). The multivariate data set is the independent variable matrix used to train the model, the prediction sample independent variable data set is assigned through the threshold of the training model, and the sample category is defined. After establishing the *A. sinensis* PLS-DA model, the classification performance of the model and the model itself were evaluated. R^2^ is the regression coefficient used in the PLS-DA model, which was used to evaluate the overall fit of the model. The recommended value of R^2^ for a good model is between 0.6–1. Q^2^ refers to the predictive ability of the model after modeling, and should be between 0.5 and 1 (Ballabio & Consonni, 2013; Albergamo et al., 2018). The Variable Importance Plot (VIP) was used to measure the variables with high contribution of the projection on the first two axes (Sayago, González-Domínguez & Beltrán, 2018). LDA is also a dimensionality reduction technique of supervised learning. LDA reduces the dimensionality of samples by maximizing the difference between groups and minimizing the difference within the group. It depends on the average within the group during dimensionality reduction. LDA and PLS-DA have the same data verification method. First, the model was verified by modeling its own data set. Due to the limited survey data, we used the leave-one-out method (LOOCV) to verify the predictive ability of the built model. Finally, statistics of real samples and model prediction samples were represented by a confusion matrix, and the model prediction accuracy rate was calculated. PCA, PLS-DA and LDA are traditional statistical discriminant methods, which usually perform well in terms of geographic origin classification when excluding redundant/confounding predictive factors (Gonzalvez, Armenta & De La Guardia, 2009). They have been widely used in the discriminant analysis of food and drug origin traceability.

The significant differences between each variable in the Linxia, Gannan and Dingxi samples and multiple comparisons of Tukey-HSD were determined using the psych package in R software (version 3.6.1). PCA was conducted using the FactoMineR and factoextra packages in R; PLS-DA model modeling, evaluation, and verification were determined by SIMCA software (version 13.0; Umetrics AB, Umeå, Sweden). The modeling and evaluation of the LDA model were conducted using SPSS (version 21.0; Chicago, USA).

## Results

### Multi-element and stable isotope characteristics of *A. sinensis*

Statistical analysis of the eight mineral elements and three stable isotope ratios of *A. sinensis* sampled from three regions were determined using ICP-MS and IRMS. Results are presented in Table 1. Among them, the K content (5639.95 ± 311.94 mg/kg) in 25 *A. sinensis* samples was the highest, and the variation range and variance were the largest, followed by Ca (869.37 ± 34.70 mg/kg), Mg (751.88 ± 34.34 mg/kg), Al (119.13 ± 10.60 mg/kg), Zn (5.32 ± 0.22 mg/kg), Mn (5.27 ± 0.22 mg/kg), Cu (1.74 ± 0.08 mg/kg) and Cr (1.02 ± 0.11 mg/kg). K, Ca, Mg, Zn, Mn and Cu were moderately variable, while Al and Cr were highly variable. The stable isotopes δ^13^C, δ^15^N and δ^18^O were - 23.79 ± 0.1‰, 2.13 ± 0.39 ‰and 25.07 ± 0.22 ‰, respectively. The highest coefficient of variation of δ^15^N is 0.91, and the coefficient of variation of δ^13^C and δ^18^O were lower than 0.1. The variation of Al, Cr and δ^15^N were highly variable. Therefore, the greater the difference between elements among *A. sinensis*, the more likely it will be suitable for origin traceability.

**Table 1:** Overview of the mineral elements and stable isotopes of *A. sinensis* from Linxia, Gannan and Dingxi.

	Range	Median	Mean	SE	std.dev	coef.var
K(mg/kg)	2806.75–8234.19	5958.66	5639.95	311.94	1559.71	0.28
Mg(mg/kg)	423.82–1191.03	748.98	751.88	34.34	171.69	0.23
Zn(mg/kg)	3.41–7.70	5.06	5.32	0.22	1.12	0.21
Cu(mg/kg)	1.02–2.55	1.67	1.74	0.08	0.39	0.22
Ca(mg/kg)	511.78–1333.02	889.09	869.37	34.7	173.52	0.2
Mn(mg/kg)	3.50–7.86	5.18	5.27	0.22	1.12	0.21
Cr(mg/kg)	0.50–2.73	0.9	1.02	0.11	0.57	0.56
Al(mg/kg)	66.38–287.77	106.49	119.13	10.6	52.99	0.44
δ^13^C(‰)	−24.83–−22.75	−23.94	−23.79	0.1	0.52	−0.02
δ^15^N(‰)	−2.08–5.57	1.91	2.13	0.39	1.95	0.91
δ^18^O(‰)	23.44–27.10	24.77	25.07	0.22	1.08	0.04

**Notes.**

Range variable range median median values from the three regions mean average values from the three regions SE standard error std.dev standard deviation coef. Var coefficient of variation

The coefficient of variation (CV) measures the degree of variation within an element (less than 0.2, has a low degree of variation; between 0.2 and 0.3, a medium variation; greater than 0.35, highly variable).

The mean ± standard error of Linxia, Gannan and Dingxi in Gansu Province were calculated to compare the differences between elements from the three regions (Table 2). Compared with the mineral elements in the three regions, Linxia *A. sinensis* K was significantly lower than Gannan and Dingxi (*P* < 0.05), while Al and Ca/Al were significantly higher than Gannan and Dingxi (*P* < 0.05). Mg, Zn, Cu, Ca, Mn, Zn/Mn and Cu/Cr had no significant difference. Among the stable isotopes, the δ^13^C and δ^15^N from Linxia were significantly (*P* < 0.05) lower than that of Gannan and Dingxi, and the δ^18^O from Gannan was significantly (*P* < 0.05) higher than that of Linxia and Dingxi.

**Table 2:** Statistical analysis of the mineral elements and stable isotopes (mean± SE) of *A. sinensis* sampled from Linxia, Gannan and Dingxi.

	LX	GN	DX
K(mg/kg)	3562.27 ± 305.41^a^	6396.38 ± 298.33^b^	6031.74 ± 401.89^b^
Mg(mg/kg)	674.73 ± 80.74^a^	689.48 ± 32.96^a^	815.15 ± 52.04^a^
Zn(mg/kg)	5.97 ± 0.76^a^	4.84 ± 0.20^a^	5.32 ± 0.29^a^
Cu(mg/kg)	2.05 ± 0.16^a^	1.54 ± 0.14^a^	1.72 ± 0.09^a^
Ca(mg/kg)	769.65 ± 86.01^a^	827.00 ± 30.92^a^	930.54 ± 52.11^a^
Mn(mg/kg)	6.13 ± 0.54^a^	5.36 ± 0.39^a^	4.90 ± 0.28^a^
Cr(mg/kg)	1.59 ± 0.32^b^	0.74 ± 0.11^a^	0.95 ± 0.14^ab^
Al(mg/kg)	186.52 ± 36.04^b^	97.98 ± 11.73^a^	104.61 ± 6.72^a^
δ^13^C(‰)	−24.39 ± 0.15^a^	−23.6 ± 0.15^b^	−23.66 ± 0.13^b^
δ^15^N(‰)	−0.15 ± 0.66^a^	3.71 ± 0.52^b^	2.17 ± 0.42^b^
δ^18^O(‰)	24.65 ± 0.30^a^	26.38 ± 0.16^b^	24.52 ± 0.23^a^

**Notes.**

Significant differences were analyzed using the ANVOA-Tukey HSD method.

Different letters indicate significant differences in the three regional variables (*P* < 0.05).

LX Linxia GN Gannan DX Dingxi

### PCA of *A. sinensis*

The original data (K, Mg, Zn/Mn, Cu/Cr, Ca/Al, δ^13^C, δ^15^N, δ^18^O) were normalized and analyzed by factor analysis. The Kaiser-Meyer-Olkin (KMO) test and Bartlett sphere test results were KMO = 0.506 (KMO > 0.5) and *P* = 0.045 (*P* < 0.05). The approximate chi-square value of Bartlett’s sphere test was 41.83, which satisfies sampling adequacy and Bartlett’s sphere test significance. Table 3 shows that in PCA analysis, the first principal component explained 38.42% of the total variance, the second principal component explained 20.31% of the total variance, and the first three principal components explained 73.68% of the total variance. These results can fully reflect the main data.

**Table 3:** Principal component analysis of the first three-axis eigenvalues and variance interpretation rate.

	Eigenvalue	Variance percent (%)	Cumulative variance percent (%)
Dim.1	2.95	38.42	38.42
Dim.2	1.56	20.31	58.73
Dim.3	1.15	14.95	73.68

**Figure 2:**
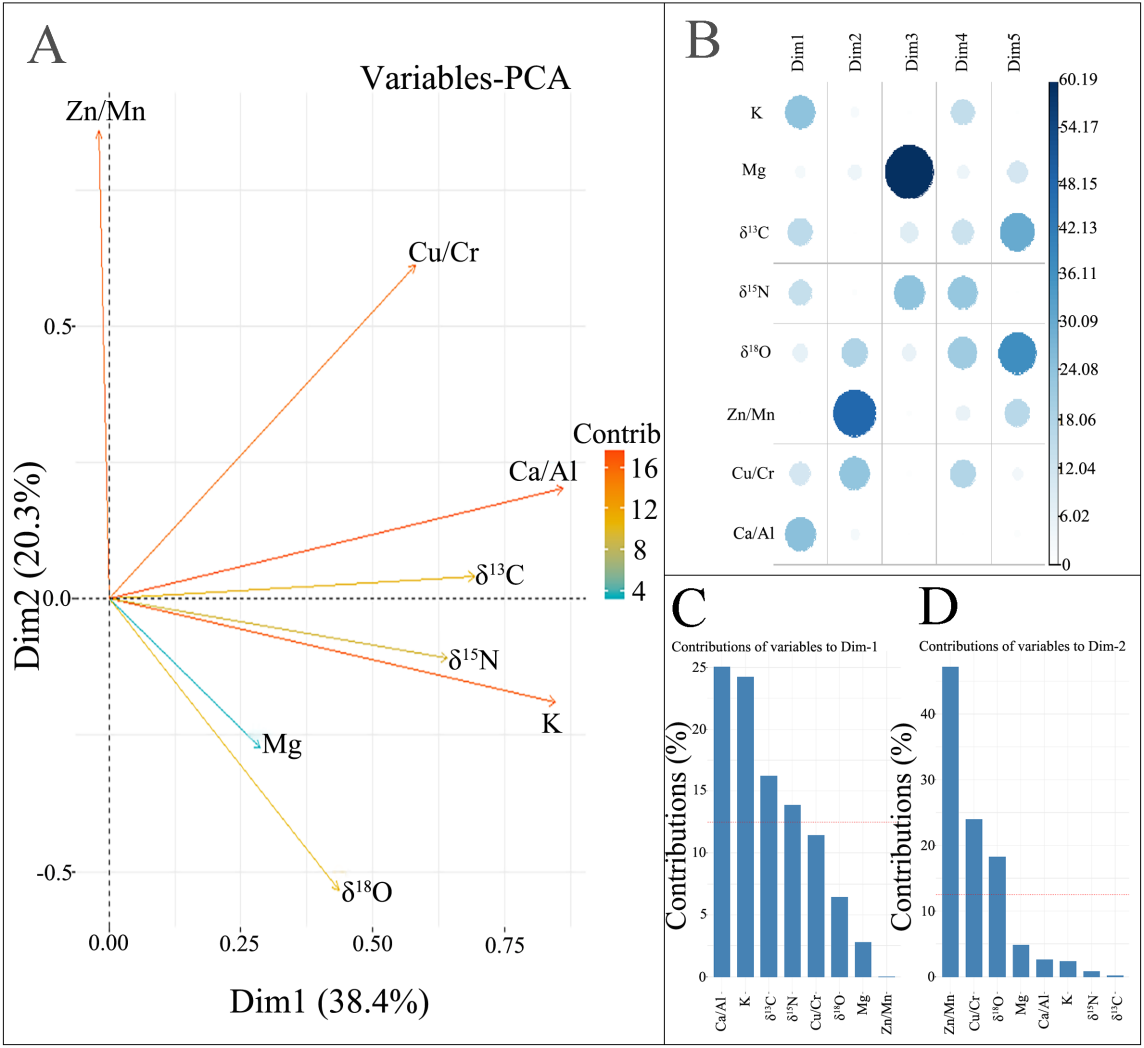
The relationship between mineral elements, stable isotopes and principal components in PCA. (A) Factor loading plot; (B) correlation plot between the principal components and each factor; (C) mineral elements and stable isotopes contributions on the first principal component axis; (D) mineral elements and stable isotopes contributions on the second principal component axis.

The relationship between the principal component and each element was explored through the factor loading plot and the contribution rate of each element on the first five principal component axes. We examined the significant correlation (*P* < 0.05) between principal component axes and mineral elements and stable isotopes (Fig. 2). Figures 2A and 2B show that Ca/Al, K, δ^13^C, and δ^15^N had a significant positive correlation (*P* < 0.05) with the first principal component axis. Zn/Mn and Cu/Cr had a significant positive correlation (*P* < 0.05) with the second principal component axis, and δ^18^O had a significant negative correlation (*P* < 0.05) with the second principal component axis. The contribution of mineral elements and stable isotopes on the first five principal component axis was explained. Ca/Al, K, δ^13^C and δ^15^N had a high contribution to the first principal component axis, while Zn/Mn, Cu/Cr and δ^18^O had a high contribution to the second principal component axis. In addition to the first two axes, the other axes have a low degree of interpretation of population variance, and K, Mg, δ^13^C, δ^15^N, δ^18^O, Zn/Mn and Cu/Cr have strong correlations in other principal component axes. Therefore, the explanations of contributions to other axes cannot be trusted. The histogram in the lower right corner of Figs. 2C and 2D quantifies the contribution of mineral elements and stable isotopes in the first two axes, and provides a threshold for the contribution of higher variables.

**Figure 3:**
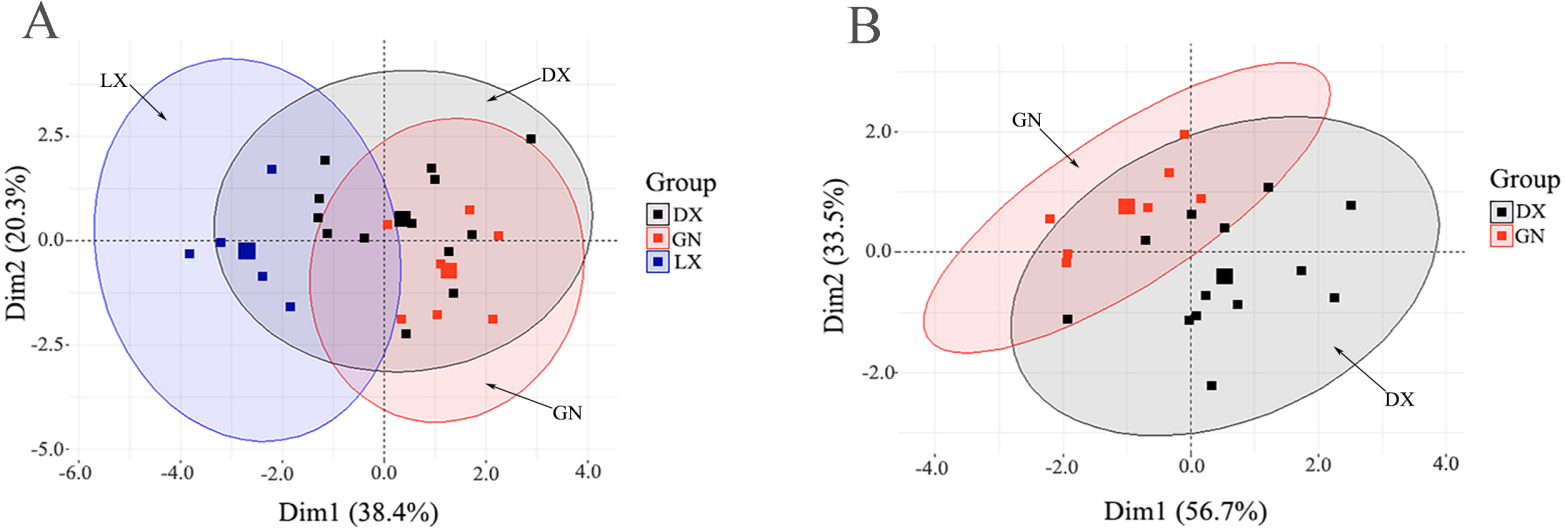
(A) *A. sinensis* sample scores from Linxia (LX), Gannan (GN) and Dingxi (DX) on the first two principal component axes; (B) *A. sinensis* sample scores from Gannan (GN) and Dingxi (DX) on the first two principal components.

The distribution of principal component scores of *A. sinensis* based on K, Mg, Zn/Mn, Cu/Cr, Ca/Al, δ^13^C, δ^15^N, and δ^18^O in the three regions on the first two principal component axes are shown in Fig. 3A. The score diagram shows that the first principal component axis can distinguish *A. sinensis* sampled from Linxia and other places. Since Ca/Al, K, δ^13^C and δ^15^N contributed more to the first principal component axis, Ca/Al, K, *δ*^13^C and δ^15^N are important factors to distinguish *A. sinensis* from Linxia and other places. It also suggests that the Ca/Al, K, δ^13^C, and δ^15^N in Linxia *A. sinensis* are lower than that of Dingxi. Although there is no clear distinction between Dingxi and Gannan, most of the samples fall into the first and fourth quadrants, respectively, suggesting that *A. sinensis* from Dingxi had higher values of Zn/Mn and Cu/Cr, while *A. sinensis* from Gannan had a higher δ^18^O value.

*A. sinensis* from Dingxi and Gannan are not easily classified (Fig. 3A). This may be due to the close geographical distance between Dingxi and Gannan. Taking into consideration that there are differences in the centroid of *A. sinensis* in Dingxi and Gannan on the second principal component axis, and that the contribution rates of Zn/Mn, Cu/Cr and δ^18^O on the second principal component axis are high, three factors of Zn/Mn, Cu/Cr and δ^18^O were used in the PCA of *A. sinensis* sampled from Dingxi and Gannan. Figure 3B shows *A. sinensis* scores from Dingxi and Gannan, and the first two axes explain 90.23% of the total variance. The first principal component axis was mainly affected by Zn/Mn and δ^18^O, and the second principal component axis was mainly affected by Cu/Cr. The samples of *A. sinensis* from Dingxi and Gannan can be clearly classified.

### PLS-DA of *A. sinensis*

We analyzed 25 samples of *A. sinensis* from Linxia, Gannan and Dingxi based on eight factors (K, Mg, Zn/Mn, Cu/Cr, Ca/Al, δ^13^C, δ^15^N, δ^18^O). Samples were analyzed using PLS-DA, and the first three component axes were extracted with eigenvalue ≥1. Table 4 shows that the first three component axes explained 70% of the total explanatory variables (the first axis explained 38%, the second axis explained 17%, and the third axis explained 15%). Fitting the response variables, the first three axes explained 68% of the total response variables (the first axis explained 29%, the second axis explained 36%, and the third axis explained 3%). In this model, the cumulative Q^2^ of the first two axes was 0.52 (suggested Q^2^ > 0.5), the negative effect of the third axis on the model’s predictive ability was slightly reduced, and the cumulative Q^2^ was 0.47.

**Table 4:** The main parameters of the first three-axis fitting of the PLS-DA model.

Component	R^2^_X_	R^2^_X_(cum)	Eigenvalue	R^2^_Y_	R^2^_Y_(cum)	Q^2^	Q^2^(cum)
1	0.38	0.38	3.06	0.29	0.29	0.19	0.19
2	0.17	0.55	1.33	0.36	0.65	0.41	0.52
3	0.15	0.70	1.21	0.03	0.68	−0.15	0.47

**Figure 4:**
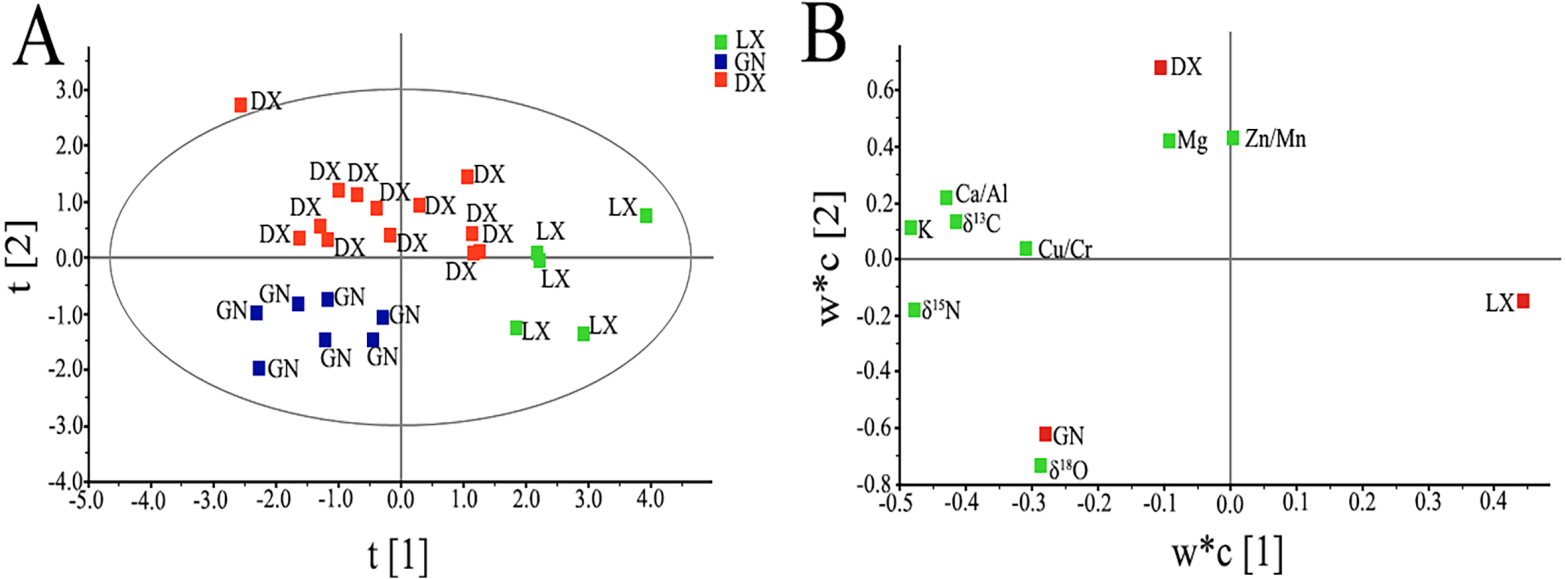
(A) PLS-DA model’s scatter score graph in different regions; (B) factor loading graph of the PLS-DA model in different regions (LX, Linxia; GN, Gannan; DX, Dingxi). In the factor load diagram, w*c [1]/[2] is used to measure the mineral elements and stable isotopes load, where w* is the weight of the standardized explanatory variable matrix obtained from the component t, and c is the weight from the component u (the weight of the response variable in the variable dimension reduction matrix). Explanatory variables near the response variable are able to distinguish data between plots.

We reduced the dimension of the explanatory variables and the scores of these variables are shown in Fig. 4A. t1 and t2 are the first and second component axes of the explanatory variables after dimensionality reduction. These explain 55% of the total variable variation. *A. sinensis* in the Linxia, Gannan and Dingxi regions are separated and clustered into one category, respectively. We were able to determine the importance of the mineral elements and stable isotopes of *A. sinensis* in PLS-DA. We also determined the correlation between variables and variables and variables and sampling areas and analyzed the PLS-DA model loading graph (Fig. 4B). K, Cu/Cr, Ca/Al, δ^13^C, and δ^15^N had larger loading values in the first component, and had a negative correlation. The samples of *A. sinensis* from Linxia were distant from the sampling areas, so these five variables were in Linxia and the samples of *A. sinensis* from Gannan and Dingxi differed. Mg, Zn/Mn, and δ^18^O had higher loading values in the second component, and the second axis was positively correlated with Mg and Zn/Mn, and negatively correlated with δ^18^O. Since the *A. sinensis* samples from Dingxi and Gannan were far apart on the second axis, these three variables are important variables for separating Dingxi and Gannan.

**Figure 5:**
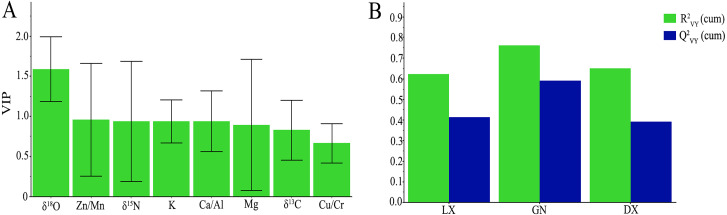
(A) The variables importance for the projection (VIP) for the *A. sinensis* PLS-DA model in different regions; (B) ${R}_{VY}^{2}$ and ${Q}_{VY}^{2}$ values in the PLS-DA cross-validation model.

We analyzed the variable component axis of the projection in the PLS-DA model, and filtered out the important variables of the projection of the entire model (Fig. 5A). In the *A. sinensis* samples, the VIPs of the five variables (δ^18^O, Zn/Mn, δ^15^N, K and Ca/Al) were all greater than 0.9 (recommended is VIP > 0.5). This indicates that these are important variables for classification in the PLS-DA model and are of great significance to the model interpretation and source traceability of *A. sinensis*.

In order to evaluate the fit and prediction performance of the cross-validation set of the built *A. sinensis* PLS-DA model, R^2^_VY_ was used to evaluate the fit of the cross-validation model, and Q^2^_VY_ was used to evaluate the predictive ability of the cross-validation model. In order to prevent the samples from overfitting in the PLS-DA model, 200 permutation tests were performed on the first three component axes of samples from Linxia, Gannan and Dingxi. These tests evaluated the validity of the cross-validation model and the stability of the model fitting. Our results show that the R^2^_VY_ of Linxia, Gannan and Dingxi were 0.62, 0.76, and 0.65, respectively, and the Q^2^_VY_ were 0.41, 0.59, and 0.39, respectively, which reflects the good fit and predictive ability of the cross-validation training set to the PLS-DA model (Fig. 5B). We applied 200 permutation tests on the first three axes of samples taken from Linxia, Gannan and Dingxi, which indicated that the built PLS-DA cross-validation model showed strong validity.

Finally, internal verification was carried out on *A. sinensis* samples from Linxia, Gannan and Dingxi based on the PLS-DA model. Due to the small data set, the original data verification and leave-one-out cross validation were used to verify the *A. sinensis* samples (Table 5). The original data successfully verified the samples of *A. sinensis* from Linxia, Gannan and Dingxi, and the accuracy rate of all the samples was 100%. We used leave-one-out cross validation to verify the samples, and the total corrected rate of all of the *A. sinensis* samples was 84%. The verification rate of *A. sinensis* from Linxia was only 60%, indicating that two samples of Linxia *A. sinensis* were wrongly classified as being from Dingxi. The verification rate of *A. sinensis* samples from Gannan was 100%, and all classifications were correct. The correct rate of the *A. sinensis* discriminant from Dingxi was 84.62%, of which 2 *A. sinensis* samples were wrongly judged as being from Linxia. This may be related to the geographical scale of Dingxi, and the large variation of variables among the species.

**Table 5:** *A. sinensis* classification results from the PLS-DA model.

Procedure	Provenance	Expected belonging groups			Correct
		LX	GN	DX	
Original	Linxia	5	0	0	100%
	Gannan	0	7	0	100%
	Dingxi	0	0	13	100%
	Total				100%
Leave-One-Out Cross Validation	Linxia	3	0	2	60%
	Gannan	0	7	0	100%
	Dingxi	2	0	11	84.62%
	Total				84%

**Notes.**

LX Linxia GN Gannan DX Dingxi

### LDA of *A. sinensis*

**Figure 6:**
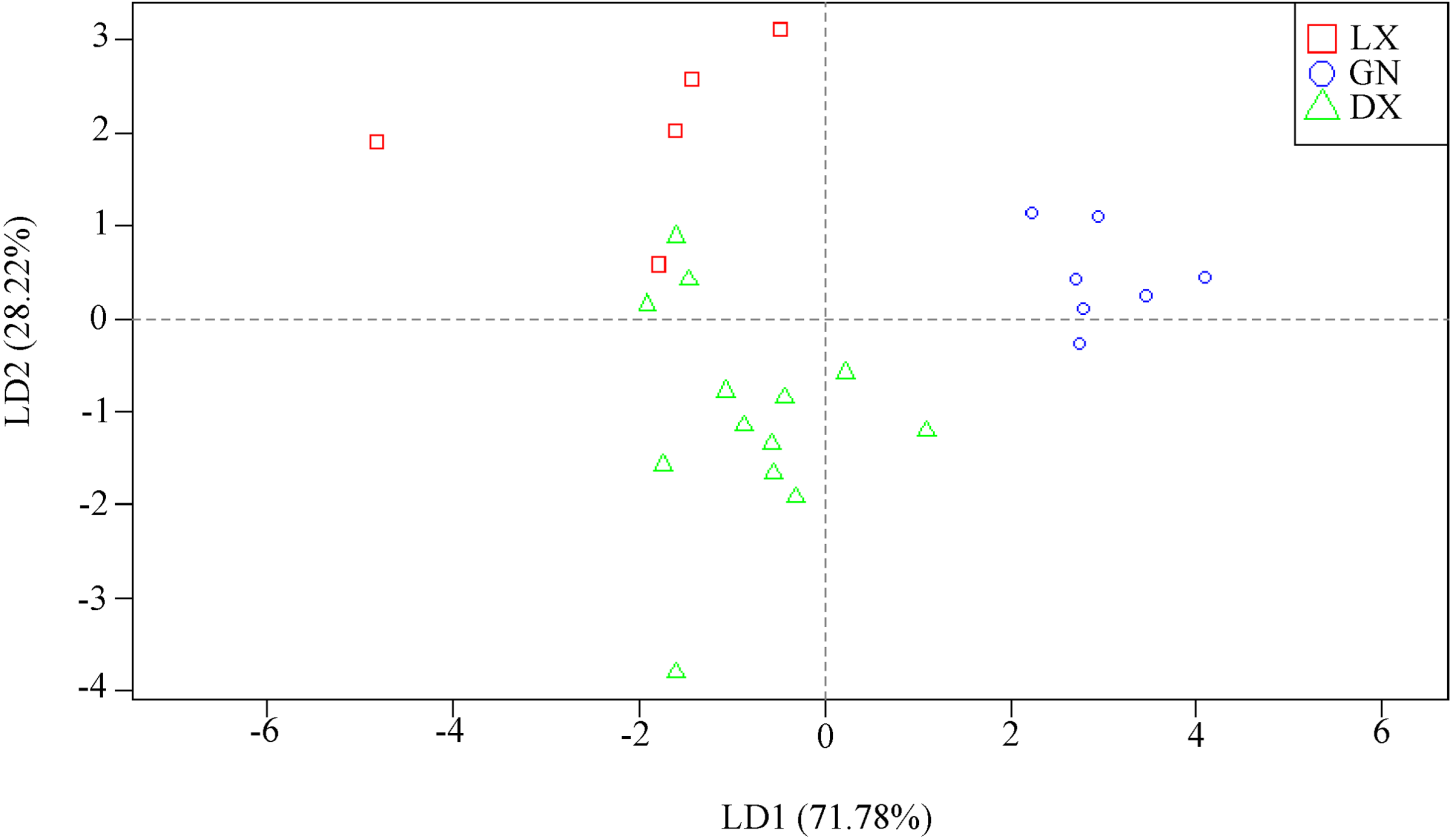
LDA model for *A. sinensis* samples from different regions (LX, Linxia; GN, Gannan; DX, Dingxi).

Stable isotopes and mineral elements were analyzed using LDA. Our analysis found that the three *A. sinensis* samples were clustered together. We also found certain differences between the groups. The recognition ability of the three stable isotopes for the two discriminant functions (LD1, LD2) explained 100% of the total variance (discrimination function 1 explained 71.8% of the total variance, and discriminant function 2 explained 28.2% of the total variance; Fig. 6). The function test was performed after the LDA model was established. The Wilks’ lambda value of the two discriminant functions was 0.073, and the difference between the groups was significant (*P* < 0.001). The discriminant functions of Linxia, Gannan and Dingxi were as follows:

Y(LX) =0.014K −0.069Mg−174.241 δ^13^C −19.921

δ^15^N+55.035 *δ*^18^O+16.287Zn/Mn+11.272Cu/Cr+5.545Ca/Al−2836.829

Y(GN) =0.014K−0.067Mg−167.540 δ^13^C−

15.833 δ^15^N+59.811 δ^18^O+11.835Zn/Mn+16.983Cu/Cr+4.480Ca/Al−2802.584

Y(DXMX) =0.014K−0.059Mg-169.654 δ^13^C-

18.146 δ^15^N+54.145 δ^18^O+18.390Zn/Mn+13.029Cu/Cr+5.614Ca/Al−2720.159

We used the original data set and the leave-one-out test set to verify the built LDA model. The accuracy of the LDA model in the original data sets of Linxia, Gannan and Dingxi was 92% (Table 6). Among them, one sample of Linxia was wrongly identified as being from Dingxi, and two sample from Dingxi were wrongly identified as being from Gannan. The correct discrimination rate was 80% using leave-one-out cross validation. The misjudgment was the same as the original data set self-validation.

**Table 6:** *A. sinensis* classification results from the LDA model.

Procedure	Provenance	Expected belonging groups			Correct
		LX	GN	DX	
Original	Linxia	4	0	1	80%
	Gannan	0	7	0	100%
	Dingxi	1	0	12	92.31%
	Total				92%
Leave-One-Out Cross Validation	Linxia	3	0	2	60%
	Gannan	0	7	0	100%
	Dingxi	2	1	10	76.92%
	Total				80%

**Notes.**

LX Linxia GN Gannan DX Dingxi

## Discussion

*A. sinensis* was discovered in Gansu and is recognized as high-quality medicinal material. In 2017, *A. sinensis* was listed as a protected product in Minxian County, Gansu. Where specimens originate plays an important role in the formation of authentic medicinal materials. Blindly introducing authentic medicinal materials may cause a decline in quality and result in fake or inferior medicinal materials used in the production and sale of medicinal products. This would in turn reduce the safety and efficacy of Chinese medicinal materials. Food and drug quality and safety issues are closely related to health and consumers rights and involve multiple interests in the entire process. Recently, frequently occuring food and drug safety incidents has aroused public attention, which affects mutual trust in trade and threatens the safety of clinical medication. It is important to address the best ways to control and assess the safety of food and drugs. Food and drug safety be monitored at every point in the food supply chain from planting and harvesting to receiving. Meanwhile, the long-term development of geographical products requires strict management, and the application of scientific and technical means for monitoring and inspection. These methods will ensure the protection of producer and consumers interests and a sustainable development of the industry. *A. sinensis* from Gansu Province requires an effective tracing method. The traceability of the authentic medicinal material will ensure control over their safety, efficacy and provenance.

Mineral elements and stable isotopes, as geographical indicators in plants, are closely related to factors such as climate, mineralogy, element mobility, bioavailability and physiological adaptability of the species. The mineral elements and stable isotopes in plants vary in different environments as determined by the plant’s adaptation to the environment and the interaction between the environment and plants. The fingerprints of mineral elements and stable isotopes in plants are the imprint of the environment. Wang et al. (2020) traced the origin of 25 mineral elements in maize from three provinces and determined that the accuracy of eight elements using the SLDA model was 92.2%. Pereira et al. (2018) traced the origin of *Arapaima spp.* fish from four different regions using strontium and carbon isotopes. Their results show that the accuracy rate of identification of wild and aquaculture fish was 58% and that of four different regions was 76%. We collected mineral elements and stable isotopes to trace the geographical origin of *A. sinensis* and determine differences between the geological and climatic environments in the three *A. sinensis*-producing areas. Based on the significant differences and the results of our model, K, Ca/Al, δ^13^C, δ^18^O and δ^15^N were determined to be important factors to distinguish *A. sinensis* in these regions.

We compared the discriminant analysis of *A. sinensis* using PLS-DA and LDA. The original data set was used to verify the model, and we found that the accuracy of the PLS-DA model was higher than that of the LDA model. The model was verified by the leave-one-out, and the accuracy rate of the PLS-DA model was higher than that of LDA model. The self-verification of the original data set to the model was a verification of the built model itself. The original data sets involved in the modeling process and the information from the verified original data set were included in the verification as the model was built. Leave-one-out cross validation is suitable for small sample analysis. A single prediction sample was removed during model modeling, the model was trained on all remaining samples, and then all samples were predicted. The total variance explained by the first two axes of the LDA model was higher than that of the PLS-DA model. Model verification using the leave-one-out method showed that PLS-DA was more correct than LDA. The PCA model is suitable for exploring the natural classification of variables in *A. sinensis* from Linxia, Gannan and Dingxi. The PLS-DA model evaluated the importance of variables and classified the samples. The PLS-DA model was also better than the LDA model in terms of classification and discrimination. Our study integrated mineral elements and stable isotopes to trace the origin of *A. sinensis* from three regions of Gansu Province. We used the PCA model to explore the data naturally, and the PLS-DA model and the LDA model to leave-one-out cross-validation. The comprehensive accuracy rates were 84% and 80%, respectively.

## Conclusion

Differences between mineral elements and stable isotopes of *A. sinensis* sampled from Linxia, Gannan and Dingxi, and Gansu Provinces were found and compared. Significant differences were found in K, Cr, Ca/Al, δ^13^C, δ^15^N, and δ^18^O (*P* < 0.05).

Significant differences were found among the three groups. PCA and PLS-DA models showed that K, Zn/Mn, Ca/Al, δ^13^C, δ^15^N and δ^18^O were important variables for distinguishing the three regions. However, only K, Ca/Al, δ^13^C, δ^18^O, and δ^15^N demonstrate significant differences among the three regions (*P* < 0.05). K, Ca/Al, δ^13^C, δ^18^O, and δ^15^N play an important role in the discriminant analysis of *A. sinensis* among the three regions.

The results of mineral elements and stable isotopes from the PLS-DA and LDA show that the discriminant accuracy rate of the PLS-DA model was 84% and the accuracy rate of the LDA model was 80%.

##  Supplemental Information

10.7717/peerj.11928/supp-1 Supplemental Information 1 Sampling coordinates

10.7717/peerj.11928/supp-2 Supplemental Information 2 The results of permutation test of *A.sinensis* in the first three axes of PLS-DA model in Dingxi area

10.7717/peerj.11928/supp-3 Supplemental Information 3 The results of permutation test of *A.sinensis* in the first three axes of PLS-DA model in Gannan area

10.7717/peerj.11928/supp-4 Supplemental Information 4 The results of permutation test of *A.sinensis* in the first three axes of PLS-DA model in Linxia area

10.7717/peerj.11928/supp-5 Supplemental Information 5 Raw Data

## Additional Information and Declarations

### Field Study Permissions

The following information was supplied relating to field study approvals (i.e., approving body and any reference numbers): Our sampling work was completed during the Angelica harvest season, and the harvest location was in the farmers’ fields. They are Xingzhen Wang, Xiaoxia Liu, Fengqiang Zhang, Baoguo Shi, and Shufen Wang. Researchers could directly contact farmers and obtain their permission to collect experimental samples.

### Data Availability

The following information was supplied regarding data availability: The data are available in the Supplemental Files.

## References

[ref-1] Adamo P, Zampella M, Quétel CR, Aversano R, Piaz FD, Tommasi ND, Frusciante L, Lorizzo M, Lepore L, Carputo D (2012). Biological and geochemical markers of the geographical origin and genetic identity of potatoes. Journal of Geochemical Exploration.

[ref-2] Ai ST, Fan XD, Fan LF, Sun Q, Liu Y, Tao XF, Dai KR (2013). Extraction and chemical characterization of *Angelica sinensis* polysaccharides and its antioxidant activity. Carbohydrate Polymers.

[ref-3] Albergamo A, Mottese AF, Bua GD, Caridi F, Sabatino G, Barrega L, Costa R, Dugo G (2018). Discrimination of the Sicilian Prickly Pear (*Opuntia Ficus-Indica* L. CV. Muscaredda) according to the provenance by testing unsupervised and supervised chemometrics. Journal of Food Science.

[ref-4] Ballabio D, Consonni V (2013). Classification tools in chemistry. Part 1: linear models. PLS-DA. Analytical Methods.

[ref-5] Bertacchini L, Cocchi M, Vigni ML, Marchetti A, Salvatore E, Sighinolfi S, Silvestri M, Durante C (2013). The impact of chemometrics on food traceability. Data Handling in Science and Technology.

[ref-6] Brereton RG, Lloyd GR (2014). Partial least squares discriminant analysis: taking the magic away. Journal of Chemometrics.

[ref-7] Brzezicha-Cirocka J, Grembecka M, Szefer P (2016). Monitoring of essential and heavy metals in green tea from different geographical origins. Environmental Monitoring and Assessment.

[ref-8] Camin F, Perini M, Bontempo L, Galeotti M, Tibaldi E, Piasentier E (2017). Stable isotope ratios of H, C, O, N and S for the geographical traceability of Italian rainbow trout (*Oncorhynchus mykiss*). Food Chemistry.

[ref-9] Catarino S, Madeira M, Monteiro F, llda C, de Sousa RB, Curvelo-Garcia A (2018). Mineral composition through soil-wine system of portuguese vineyards and its potential for wine traceability. Beverages.

[ref-10] Catarino S, Madeira M, Monteiro F, Rocha F, Curvelo-Garcia AS, De Sousa RB (2008). Effect of bentonite characteristics on the elemental composition of wine. Journal of Agricultural & Food Chemistry.

[ref-11] Chao WW, Lin BF (2011). Bioactivities of major constituents isolated from Angelica sinensis (Danggui). Chinese Medicine.

[ref-12] Chen TB, Ding KF, Hao SK, Li GD, Qu JY (2020). Batch-based traceability for pork: a mobile solution with 2D barcode technology. Food Control.

[ref-13] Circosta C, De Pasquale R, Palumbo DR, Samperi S, Occhiuto F (2006). Estrogenic activity of standardized extract of *Angelica sinensis*. Phytotherapy Research.

[ref-14] Coelho I, Matos AS, Teixeira R, Nascimento A, Bordado J, Donard O, Castanheira I (2019). Combining multielement analysis and chemometrics to trace the geographical origin of Rocha pear. Journal of Food Composition and Analysis.

[ref-15] Damak F, Asano M, Baba K, Suda A, Araoka D, Wali A, Isoda H, Nakajima M, Ksibi M, Tamura K (2019). Interregional traceability of Tunisian olive oils to the provenance soil by multielemental fingerprinting and chemometrics. Food Chemistry.

[ref-16] Deng XF, Liu Z, Zhan Y, Ni K, Zhang YZ, Ma WZ, Shao SZ, Lv XN, Yuan YW, Rogers KM (2019). Predictive geographical authentication of green tea with protected designation of origin using a random forest model. Food Control.

[ref-17] Fang S, Huang WJ, Wei YM, Tao M, Hu X, Li TH, Kalkhajeh YK, Wei W (2019). Geographical origin traceability of Keemun black tea based on its non-volatile composition combined with chemometrics. Journal of the Science of Food and Agriculture.

[ref-18] Ferrio JP, Voltas J, Araus JL (2003). Use of carbon isotope composition in monitoring environmental changes. Management of Environmental Quality an International Journal.

[ref-19] Geană E, Sandru C, Stanciu V, Ionete RE (2017). Elemental profile and ^87^Sr/^86^Sr isotope ratio as fingerprints for geographical traceability of wines: an approach on Romanian Wines. Food Analytical Methods.

[ref-20] George RV, Harsh HO, Ray P, Babu AK (2019). Food quality traceability prototype for restaurants using blockchain and food quality data index. Journal of Cleaner Production.

[ref-21] Giacomelli N, Yang YP, Huber FK, Ankli A, Weckerle CS (2017). Angelica sinensis (Oliv.) Diels: influence of value chain on quality criteria and marker compounds Ferulic Acid and Z-Ligustilide. Medicine.

[ref-22] Gonzalvez A, Armenta S, De La Guardia M (2009). Trace-element composition and stable-isotope ratio for discrimination of foods with Protected Designation of Origin. Trends in Analytical Chemistry.

[ref-23] Granath U, Rydin H, Baltzer JL, Bengtsson F, Boncek N, Bragazza L, Bu ZJ, Caporn Simon JM, Dorrepaal E, Galanina O, Gałka M, Ganeva A, Gillikin DP, Goia I, Goncharova N, Hájek M, Haraguchi A, Harris LI, Humphreys E, Jiroušek M, Kajukało K, Karofeld E, Koronatova NG, Kosykh NP, Lamentowicz M, Lapshina E, Limpens J, Linkosalmi M, Ma JZ, Mauritz M, Mun TM (2018). Environmental and taxonomic controls of carbon and oxygen stable isotope composition in *Sphagnum* across broad climatic and geographic ranges. Biogeoences.

[ref-24] Greenough JD, Mallory-Greenough LM, Fryer BJ (2005). Geology and Wine 9: regional trace element fingerprinting of Canadian wines. Geoscience Canada.

[ref-25] Han C, Dong SL, Li L, Wei FY, Zhou YG, Gao QF (2020). The effect of the seasons on geographical traceability of salmonid based on multi-element analysis. Food Control.

[ref-26] Handley LL, Raven JA (2010). The use of natural abundance of nitrogen isotopes in plant physiology and ecology. Plant Cell & Environment.

[ref-27] Hao LZ, Yang X, Huang YY, Hocquette JF, Liu SJ (2019). Using mineral elements to authenticate the geographical origin of Yak Meat. Kafkas Universitesi Veteriner Fakultesi Dergisi.

[ref-28] Hook Ingrid LI (2014). Danggui to *Angelica sinensis* root: are potential benefits to European women lost in translation? A review. Journal of Ethnopharmacology.

[ref-29] Hu XZ, Liu SQ, Li XH, Wang CX, Xu CH (2019). Geographical origin traceability of Cabernet Sauvignon wines based on Infrared fingerprint technology combined with chemometrics. Scientific Reports.

[ref-30] Hultine KR, Marshall JD (2000). Altitude trends in conifer leaf morphology and stable carbon isotope composition. Oecologia.

[ref-31] Innamorato V, Longobardi F, Lippolis V, Cortese M, Logrieco AF (2019). Tracing the geographical origin of Lentils (*Lens culinaris Medik*.) by infrared spectroscopy and chemometrics. Food Analytical Methods.

[ref-32] Jin ML, Zhao K, Huang QS, Shang PXuCL (2012). Isolation, structure and bioactivities of the polysaccharides from *Angelica sinensis* (Oliv.) Diels: a review. Carbohydrate Polymers.

[ref-33] Li L, Cui H, Dong SL, Boyd CE (2018). Use of elemental profiling and isotopic signatures to differentiate Pacific white shrimp (*Litopenaeus vannamei*) from freshwater and seawater culture areas. Food Control.

[ref-34] Liu Y, Zhang XF, Li Y, Wang HX (2017). The application of compound-specific isotope analysis of fatty acids for traceability of sea cucumber (*Apostichopus japonicus*) in the coastal areas of China. Journal of the Science of Food and Agriculture.

[ref-35] Liu Z, Zhang WX, Zhang YZ, Chen TJ, Rogers KM (2018). Assuring food safety and traceability of polished rice from different production regions in China and Southeast Asia using chemometric models. Food Control.

[ref-36] Lu GH, Chan K, Leung K, Chan CL, Zhao ZZ, Jiang ZH (2005). Assay of free ferulic acid and total ferulic acid for quality assessment of *Angelica sinensis*. Journal of Chromatography A.

[ref-37] Lu CC, Liu M, Shang WR, Yuan Y, Yang KH (2020). Knowledge mapping of *Angelica sinensis* (Oliv.) Diels (Danggui) research: a scientometric study. Frontiers in Pharmacology.

[ref-38] Lukić I, Carlin S, Horvat I, Vrhovsek U (2018). Combined targeted and untargeted profiling of volatile aroma compounds with comprehensive two-dimensional gas chromatography for differentiation of virgin olive oils according to variety and geographical origin. Food Chemistry.

[ref-39] Luo RJ, Jiang T, Chen XB, Zheng CC, Liu HB, Yang J (2019). Determination of geographic origin of Chinese mitten crab (*Eriocheir sinensis*) using integrated stable isotope and multi-element analyses. Food Chemistry.

[ref-40] Mei ZQ, Zhang C, Khan MA, Zhu Y, Tania M, Luo PY, Fu JJ (2015). Efficiency of improved RAPD and ISSR markers in assessing genetic diversity and relationships in *Angelica sinensis* (Oliv.) Diels varieties of China. Electronic Journal of Biotechnology.

[ref-41] Pepi S, Chicca M, Telloli C, Di Roma A, Grisenti P, Tessari U, Vaccaro C (2019). Discrimination of geographical origin of hop (*Humulus lupulus* L.) using geochemical elements combined with statistical analysis. Environmental Geochemistry and Health.

[ref-42] Pereira LA, Santos RV, Hauser M, Duponchelle F, Carvajai F, Pecheyran C, Berail S, Pouilly M (2018). Commercial traceability of Arapaima spp. fisheries in the Amazon Basin: can biogeochemical tags be useful?. Biogeoences.

[ref-43] Pianezze S, Perini M, Bontempo L, Ziller L, D’Archivio AA (2019). Geographical discrimination of garlic (*Allium Sativum* L.) based on Stable isotope ratio analysis coupled with statistical methods: the Italian case study. Food and Chemical Toxicology.

[ref-44] Potortì AG, Bella GD, Mottese AF, Bua GD, Fede MR, Sabatino G, Salvo A, Somma R, Dugo G, Turco VL (2018). Traceability of Protected Geographical Indication (PGI) Interdonato lemon pulps by chemometric analysis of the mineral composition. Journal of Food Composition and Analysis.

[ref-45] Ripullone F, Matsuo N, Stuart-Williams H, Wong SC, Borghetti M, Tani M, Farquhar G (2008). Environmental effects on oxygen isotope enrichment of leaf water in cotton leaves. Plant Physiology.

[ref-46] Ross IA (2001). Angelica sinensis L. Medicinal plants of the world.

[ref-47] Sayago A, González-Domínguez R, Beltrán R (2018). Combination of complementary data mining methods for geographical characterization of extra virgin olive oils based on mineral composition. Food Chemistry.

[ref-48] Tang Q, Li JJ, Sun M, Lv J, Gai RY, Mei L, Xu LZ (2015). Food traceability systems in China: the current status of and future perspectives on food supply chain databases, legal support, and technological research and support for food safety regulation. BioScience Trends.

[ref-49] Tian SY, Hao CC, Xu GK, Yang JJ, Sun RG (2017). Optimization conditions for extracting polysaccharide from *Angelica sinensis* and its antioxidant activities. Journal of Food & Drug Analysis.

[ref-50] Wadood SA, Guo BL, Wei YM (2019). Geographical traceability of wheat and its products using multielement light stable isotopes coupled with chemometrics. Journal of Mass Spectrometry.

[ref-51] Wang YY, Li JQ, Liu HG, Wang YZ (2019). Attenuated Total Reflection-Fourier Transform Infrared Spectroscopy (ATR-FTIR) combined with chemometrics methods for the classification of Lingzhi Species. Molecules.

[ref-52] Wang F, Zhao HY, Yu CD, Tang J, Wu W, Yang QL (2020). Determination of the geographical origin of maize (*Zea mays* L.) using mineral element fingerprints. Journal of the Science of Food and Agriculture.

[ref-53] Wu H, Tian L, Chen B, Jin BH, Lin GH (2019). Verification of imported red wine origin into China using multi isotope and elemental analyses. Food Chemistry.

[ref-54] Zhang J, Yang RD, Chen R, Li YC, Peng YS, Liu CL (2018). Multielemental analysis associated with chemometric techniques for geographical origin discrimination of tea leaves (*Camelia sinensis*) in Guizhou Province, SW China. Molecules.

[ref-55] Zhao KJ, Dong Tina TX, Tu PF, Song ZH, Tsim Karl WK (2003). Molecular genetic and chemical assessment of radix *Angelica* (Danggui) in China. Journal of Agricultural and Food Chemistry.

[ref-56] Zhou P, Li ZY, Ouyang LQ, Gong XD, Meng P, Dai M, Wang Z, Wang Y (2019). A multi-element stable isotope approach coupled with chemometrics for the determination of Tieguanyin tea geographical origin and harvest season. Analytical Methods.

[ref-57] Zhao HY, Zhang SL, Zhang ZW (2017). Relationship between multi-element composition in tea leaves and in provenance soils for geographical traceability. Food Control.

