# Supplementary figures and images for: Validation and analysis of the geographical origin of Angelica sinensis (Oliv.) Diels using multi-element and stable isotopes
Source: PeerJ. 2021 Aug 6;9:e11928. doi: 10.7717/peerj.11928 (PMC8351574; doi:10.7717/peerj.11928)

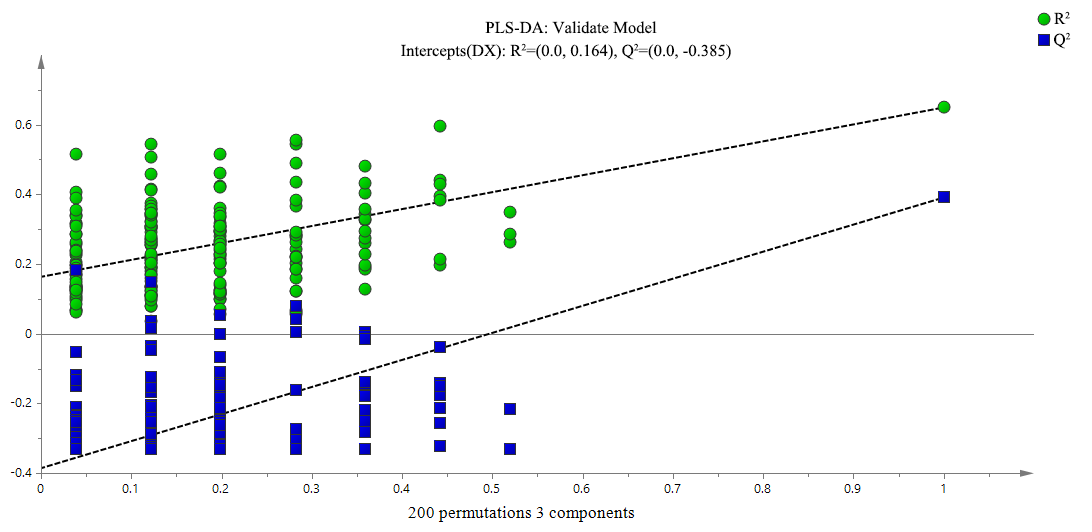

Supplement: Supplemental Information 2 [file peerj-09-11928-s002.png]

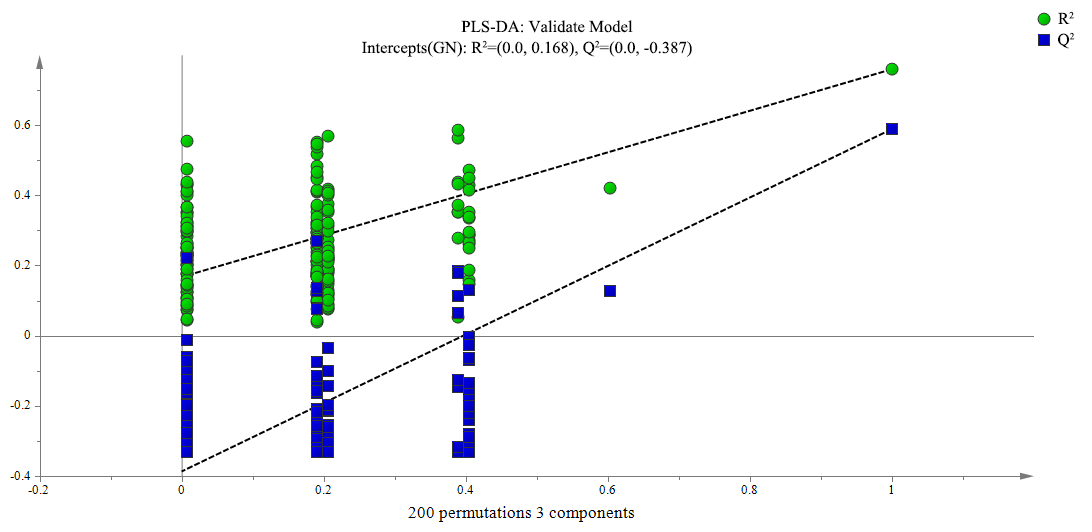

Supplement: Supplemental Information 3 [file peerj-09-11928-s003.png]

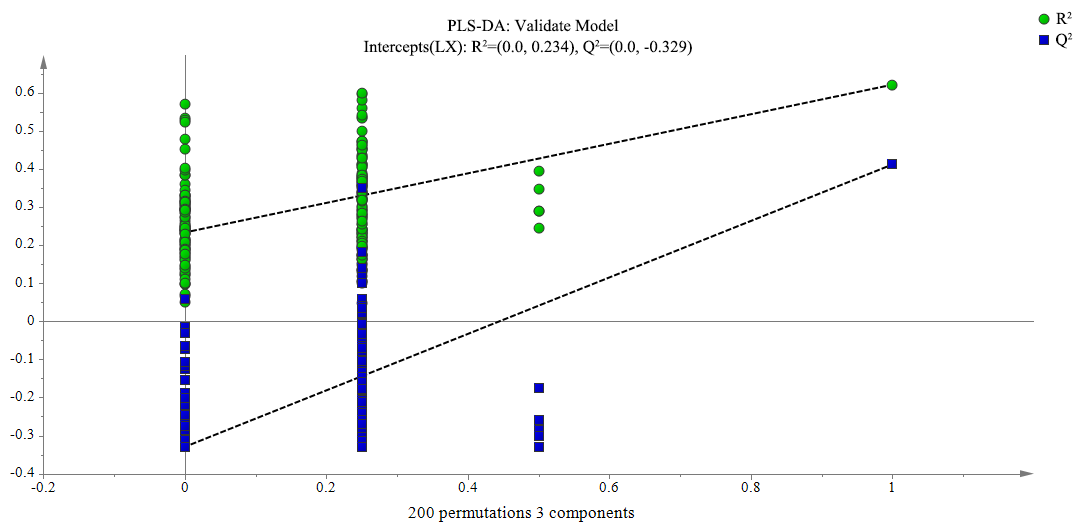

Supplement: Supplemental Information 4 [file peerj-09-11928-s004.png]
